# Digital Technologies as a Means to Reducing Mental Health Disparities: The Role of Ethnicity, SES, and Geography

**DOI:** 10.1093/geroni/igaa057.2291

**Published:** 2020-12-16

**Authors:** Giyeon Kim

**Affiliations:** Chung-Ang University, Seoul, Republic of Korea

## Abstract

This presentation discusses the importance of using digital technologies on reducing mental health disparities among older adults from diverse backgrounds. This talk primarily focuses on the role of ethnicity, socioeconomic status and geography. First, the speaker presents the current status of digital technology use among older adults and how different levels of digital technology use affect mental health disparities by ethnicity, SES, and place of residence. Second, the speaker introduces a recently funded government project on developing an IoT-based home system (Internet of Things) to screen mild cognitive function for Korean older adults. Lastly, the speaker discusses potential implications, as well as directions for future research on using digital technologies to reduce mental health disparities among diverse populations.

